# Supplementary material for: Native and invasive squirrels show different behavioural responses to scent of a shared native predator
Source: R Soc Open Sci. 2020 Feb 26;7(2):191841. doi: 10.1098/rsos.191841 (PMC7062111; doi:10.1098/rsos.191841)
Supplement: Site information [file rsos191841supp1.docx]

# **9. SUPPLEMENTARY INFORMATION**

**Table S1.** Habitat, squirrel species present, and latitude and longitude of sites for behavioural study.

| **Site** | **Habitat** | **Species** | **Lat** | **Long** |
| --- | --- | --- | --- | --- |
| Tollymore 01 | Mixed | Red | 54.22243 | -5.9465 |
| Tollymore 02 | Mixed | Red | 54.22181 | -5.93245 |
| Nugent’s Woods 01 | Mixed | Red | 54.39861 | -5.54563 |
| Nugent’s Woods 02 | Mixed | Red | 54.38824 | -5.54998 |
| Ring of Gullion 01 | Coniferous Plantation | Red | 54.09322 | -6.48071 |
| Ring of Gullion 02 | Coniferous Plantation | Red | 54.10786 | -6.44473 |
| Ballywalter Garden | Suburban Garden | Red | 54.53848 | -5.49855 |
| Mount Stewart 01 | Mixed | Red | 54.56377 | -5.59164 |
| Mount Stewart 02 | Mixed | Red | 54.56427 | -5.6095 |
| Ballywhite | Coniferous Plantation | Red | 54.40129 | -5.56831 |
| Garranard | Suburban Garden | Grey | 54.60574 | -5.86136 |
| Balmoral | Suburban Garden | Grey | 54.56427 | -5.9534 |
| Bristow Park | Mixed | Grey | 54.55939 | -5.96186 |
| Danesford | Suburban Garden | Grey | 54.57109 | -5.94143 |
| Dunmurry | Deciduous Forest | Grey | 54.54748 | -6.01275 |
| Lacefield | Suburban Garden | Grey | 54.59457 | -5.84986 |
| Newforge | Suburban Garden | Grey | 54.55715 | -5.94771 |
| Stranmillis | Deciduous Forest | Grey | 54.57255 | -5.93302 |
| Torrosch | Mixed | Grey | 54.59239 | -5.77495 |
| UFTM | Mixed | Grey | 54.65099 | -5.79754 |
