# Supplementary material for: Native and invasive squirrels show different behavioural responses to scent of a shared native predator
Source: R Soc Open Sci. 2020 Feb 26;7(2):191841. doi: 10.1098/rsos.191841 (PMC7062111; doi:10.1098/rsos.191841)
Supplement: Behaviour breakdown [file rsos191841supp2.docx]

**Table S2.** Full breakdown of behaviours of both squirrel species 2 days pre and post treatment of feeder with pine marten scent.

| **Site** | **Species** | **Treatment** | **Feeding (%)** | **Vigilance (%)** | **Movement (%)** | **Investigatory (%)** | **Aggression (%)** | **Social (%)** |
| --- | --- | --- | --- | --- | --- | --- | --- | --- |
| DF | Grey Squirrel | Post | 94.28021 | 0.578406 | 0.514139 | 3.727506 | 0.899743 | 0 |
| DF | Grey Squirrel | Pre | 95.16908 | 3.751066 | 0.511509 | 0.426257 | 0.142086 | 0 |
| DM | Grey Squirrel | Pre | 83.57581 | 11.76404 | 0.466015 | 1.977722 | 2.216413 | 0 |
| DM | Grey Squirrel | Post | 90.6501 | 6.790585 | 0.382963 | 0.737904 | 1.438446 | 0 |
| NF | Grey Squirrel | Pre | 86.40916 | 8.583691 | 0 | 4.864092 | 0.143062 | 0 |
| NF | Grey Squirrel | Post | 90.50847 | 4.067797 | 0.338983 | 4.915254 | 0.169492 | 0 |
| NW01 | Red Squirrel | Pre | 81.13208 | 0 | 0 | 16.19497 | 2.672956 | 0 |
| NW01 | Red Squirrel | Post | 0 | 0 | 0 | 0 | 0 | 0 |
| NW02 | Red Squirrel | Pre | 92.87289 | 5.948875 | 0.129579 | 1.036636 | 0.012016 | 0 |
| NW02 | Red Squirrel | Post | 61.71171 | 29.61712 | 0.187688 | 8.483483 | 0 | 0 |
| TM02 | Red Squirrel | Pre | 90.80363 | 7.973001 | 0.021093 | 0.727695 | 0.474583 | 0 |
| TM02 | Red Squirrel | Post | 0 | 0 | 0 | 0 | 0 | 0 |
| UFTM | Grey Squirrel | Pre | 95.20484 | 4.120112 | 0.046555 | 1.040611 | -0.41212 | 0 |
| UFTM | Grey Squirrel | Post | 93.30192 | 4.15685 | 0 | 1.935375 | 0.605857 | 0 |
| BWG | Red Squirrel | Pre | 91.19207 | 8.13307 | 0.005487 | 0 | 0.669373 | 0 |
| BWG | Red Squirrel | Post | 73.83513 | 25.98566 | 0 | 0 | 0.179207 | 0 |
| GN | Grey Squirrel | Pre | 97.32728 | 2.033409 | 0.028872 | 0.338214 | 0.272221 | 0 |
| GN | Grey Squirrel | Post | 98.0874 | 1.59787 | 0 | 0.314732 | 0 | 0 |
| LF | Grey Squirrel | Pre | 81.19033 | 13.96221 | 0.157033 | 3.191131 | 1.439468 | 0.059822 |
| LF | Grey Squirrel | Post | 93.90333 | 5.469746 | 0.163178 | 0.279935 | 0.33339 | 0.130355 |
| MS03 | Red Squirrel | Pre | 97.49878 | 2.110786 | 0.024402 | 0.073206 | 0.292826 | 0 |
| MS03 | Red Squirrel | Post | 0 | 0 | 0 | 0 | 0 | 0 |
| MS04 | Red Squirrel | Pre | 90.0448 | 2.289696 | 0.149328 | 7.665505 |  | 0 |
| MS04 | Red Squirrel | Post | 0 | 0 | 0 | 0 |  | 0 |
| SG02 | Red Squirrel | Pre | 98.47032 | 0 | 0 | 1.347032 | 0 | 0 |
| SG02 | Red Squirrel | Post | 86.88398 | 10.27977 | 0 | 1.899384 |  | 0 |
| SG01 | Red Squirrel | Pre | 98.34402 | 1.603613 | 0.039272 | 0.013091 |  | 0 |
| SG01 | Red Squirrel | Post | 95.36352 | 3.29347 | 0.028474 | 0.313212 | 0 | 0.873197 |
| BM | Grey Squirrel | Pre | 97.79638 | 0.93097 | 0 | 0 | 0 | 0 |
| BM | Grey Squirrel | Post | 96.8661 | 0 | 0 | 0 | 0 | 0 |
| TS | Grey Squirrel | Pre | 99.77578 | 0 | 0 | 0 | 0 | 0 |
| TS | Grey Squirrel | Post | 100 | 0 | 0 | 0 | 0 | 0 |
| BW | Red Squirrel | Pre | 89.34547 | 10.98261 | 0 | 0 | 0 | 0 |
| BW | Red Squirrel | Post | 83.68449 | 17.20078 | 0 | 0 | 0 | 0 |
